# Cannabinoids in the Treatment of Cannabis Use Disorder: Systematic Review of Randomized Controlled Trials

**DOI:** 10.3389/fpsyt.2022.867878

**Published:** 2022-06-22

**Authors:** Caroline Vuilleumier, Norbert Scherbaum, Udo Bonnet, Patrik Roser

**Affiliations:** ^1^Department of Psychiatry and Psychotherapy, Psychiatric Services Aargau, Academic Teaching Hospital of the University of Zurich, Windisch, Switzerland; ^2^Department of Psychiatry and Psychotherapy, LVR-Hospital Essen, Medical Faculty, University of Duisburg Essen, Essen, Germany; ^3^Department of Psychiatry, Psychotherapy and Psychosomatic Medicine, Evangelisches Krankenhaus Castrop-Rauxel, Academic Teaching Hospital of the University of Duisburg-Essen, Castrop-Rauxel, Germany

**Keywords:** cannabis use disorder (CUD), cannabinoids, treatment, randomized controlled trial, endocannabinoid system (ECS), efficacy and safety

## Abstract

**Background:**

The prevalence of cannabis use and cannabis use disorders (CUD) has significantly increased over time. However, there are no approved pharmacological treatments for CUD. The aim of this study was to determine the efficacy and safety of various medical cannabinoids in the treatment of CUD.

**Methods:**

We conducted a systematic review of randomized controlled trials which evaluated the therapeutic potential of medical cannabinoids in individuals with CUD and summarized the main study outcomes in terms of cannabis use, abstinence, withdrawal symptoms, craving, retention in treatment and adverse events.

**Results:**

We identified eight trials with a total of 667 study participants. Dronabinol reduced cannabis withdrawal symptoms whereas nabiximols, cannabidiol and PF-04457845, a fatty acid amide inhibitor, also reduced cannabis use and improved abstinence, compared to placebo. Nabilone failed to demonstrate efficacy in the treatment of CUD. All medications were well-tolerated.

**Conclusions:**

Cannabinoid receptor agonists, i.e., dronabinol and nabilone, showed only limited or no therapeutic potential in the treatment of CUD. In contrast, modulators of endocannabinoid activity, i.e., nabiximols, cannabidiol and PF-04457845, demonstrated broader efficacy which covered almost all aspects of CUD. Endocannabinoid modulation appears to be a promising treatment approach in CUD, but the evidence to support this strategy is still small and future research in this direction is needed.

## Introduction

To date, cannabis is still the most widely used illicit drug worldwide, although meanwhile legalized for recreational purposes in several countries, with, in 2019, almost 4% of the global population (aged 15 to 64 years) having used cannabis at least once, the equivalent of about 200 million people (1). In Central and Western Europe as well as North America, the risk perception associated with cannabis use is on the decrease, while regular cannabis use increased in the long-term, with a prevalence of 7.8 and 14.5%, respectively, in the adult population (1).

This development poses a notable public health issue as recreational cannabis use is associated with considerable adverse health effects, including cognitive deficits, motor impairment and psychosis (2). In addition, about 20–30% of the regular cannabis users have been found to develop a cannabis use disorder (CUD) over time (3). The risk of CUD increases with daily cannabis use, earlier age at first use and higher potency of cannabis (4). According to the Diagnostic and Statistical Manual of Mental Disorders, 5^th^ Edition (DSM-V), CUD is defined by impaired control, social impairment, risky use, tolerance, and withdrawal (5). A recent meta-analysis reported that almost half of the regular or dependent cannabis users is affected by a cannabis withdrawal syndrome (6) which typically begins soon after cessation of use, peaks within a couple of days and lasts for up to 3 weeks (7). The cannabis withdrawal syndrome is characterized by craving, irritability, nervousness, sleep disorders, depressed mood and decreased appetite (8).

Although the number of people with CUD seeking treatment is increasing in Europe and North America, the overall utilization of CUD-specific treatments is relatively low and the majority of the affected individuals is untreated (9, 10). In the United States, CUD treatment seeking behavior and CUD treatment admissions among young adults (aged 18 to 24 years) have even declined (11). On the other hand, effective treatment options for CUD are limited and focus primarily on psychosocial interventions, including motivational enhancement therapy, cognitive behavioral therapy and contingency management (12). However, access to psychosocial interventions as well as coverage from insurance companies are often limited. Moreover, clinical trials showed that these treatments are cost-intensive and their abstinence rates are only modest and decline after treatment, raising the need for further therapeutic options, especially pharmacological treatments (13–16).

In the last two decades, numerous studies explored the potential of different medications with various pharmacological targets for the treatment of CUD. Human laboratory and clinical studies particularly aimed to identify pharmaceutical agents which are effective in the treatment of cannabis withdrawal syndromes, the maintenance of abstinence, the retention in treatment and the reduction of cannabis use. The most promising candidates included several antidepressants (e.g., bupropion, escitalopram, mirtazapine, nefazodone and venlafaxine), antipsychotics (e.g., quetiapine), anticonvulsants (e.g., valproic acid, gabapentin and topiramate) as well as lithium, buspirone and N-acetylcysteine (17, 18). Although some of these agents produced some benefits for distinct individual aspects in patients with CUD, none of these treatments has demonstrated sufficient empirical evidence to provide clear therapeutic recommendations and to achieve the approval for the treatment of CUD by the authorities, mainly due to insufficient study designs, sample sizes and outcome measures (19).

More recently, the endocannabinoid system and its components have been proposed to provide novel and unique systemic targets for the treatment of CUD. The primary constituent of cannabis, Δ^9^-tetrahydrocannabinol (THC), produces its acute psychoactive effects via partial agonism at the cannabinoid type 1 receptor (CB1) in the central nervous system (20). Regular cannabis use is associated with the development of craving, tolerance and withdrawal symptoms which was related to a dysregulation of the endocannabinoid system, particularly to CB1 receptor downregulation and desensitization and reduced levels of the endocannabinoids N-arachidonoylethanolamide (AEA) and 2-arachidonoylglycerol (2-AG) (21). It was therefore suggested that the potentiation of endocannabinoid function by medical cannabinoids might serve as a promising treatment strategy for CUD (22). In this context, the following cannabinoids and cannabinoid preparations are of particular interest: dronabinol (THC), nabilone, a synthetic derivative of THC, cannabidiol (CBD), a natural inhibitor of the hydrolysis and reuptake of endocannabinoids as well as a negative allosteric modulator of the CB1 receptor, nabiximols, which contains a combination of THC and CBD at a ratio of ~1:1, and PF-04457845, a synthetic inhibitor of the endocannabinoid-degrading enzyme fatty acid amide hydrolase (FAAH).

This systematic review aims to summarize and discuss the main findings of randomized controlled trials (RCTs) evaluating the efficacy, safety and tolerability of different medical cannabinoids in the treatment of CUD.

## Methods

### Information Sources and Search

Following the Preferred Reporting Items for Systematic Reviews and Meta-Analyses (PRISMA) guidelines (23), we systematically searched the PubMed/Medline database on November 6th, 2021, to identify all relevant studies. We also checked the reference lists of included studies and previous reviews. We used the following search terms: (cannabis OR marijuana OR marihuana OR THC OR tetrahydrocannabinol) AND (dependence OR withdrawal OR craving OR relapse) AND (treatment OR therapy OR medication OR replacement) AND (dronabinol OR nabilone OR nabiximols OR sativex OR cesamet OR FAAH OR fatty acid amide hydrolase OR CBD OR cannabidiol).

### Eligibility Criteria

We defined the eligibility criteria following the Population-Intervention-Comparison-Outcomes-Study Design (PICOS) model (24):

- Population: adults (aged 18 years or older) with a diagnosis of cannabis use disorder (CUD) according to a valid diagnostic classification system, e.g., ICD-10 or DSM-V.
- Intervention: any pharmacotherapy with medical cannabinoids, i.e., dronabinol, nabilone, nabiximols, cannabidiol or endocannabinoid modulators, as monotherapy or in combination with another medication, with or without concomitant psychotherapy.
- Comparison: placebo.
- Outcomes: reduction of cannabis use, maintenance of abstinence, reduction of withdrawal symptoms, reduction of craving, retention in treatment, safety, and tolerability.
- Study Design: randomized controlled studies.

Review articles, guidelines, expert opinions, study protocols, commentaries as well as human experimental and animal studies were excluded from this review.

### Study Selection

At first, the titles and, if necessary, abstracts of all records were independently screened by two authors (CV, PR) and those articles which did not meet the eligibility criteria were excluded. Afterwards, the same two authors independently read the remaining articles in full-text which again were checked for the above-mentioned eligibility criteria. Only those articles which met the final eligibility criteria were included to the systematic review. All discrepancies were resolved through discussion and consensus.

### Data Collection and Data Items

The same two authors extracted all relevant data from the included studies and abstracted the following items according to the PICOS model:

- Population: sample size, mean age, sex, cannabis use characteristics.
- Intervention: medical cannabinoid, dosage regimen, additional medication (if applicable), concomitant psychotherapy.
- Comparison: placebo regimen.
- Outcomes: measures of cannabis use (in grams or joints per day or week), duration of abstinence, measures of withdrawal symptoms, measures of craving, duration of retention in treatment, adverse events.
- Study Design: first author, year of publication, trial location, study characteristics (setting, duration, follow-up).

### Risk of Bias Assessment

We assessed the risk of bias in every individual trial by using the Cochrane Collaboration's Risk of Bias Tool in randomized controlled trials (25) and assigned a rating of “low,” “high” or “unclear” risk to each of the seven bias domains (randomization, allocation concealment, participant blinding, researcher blinding, selective reporting, attrition and other risks of bias). Based on the number of domains classified as “low risk,” we also created an “overall” risk of bias. The risk of bias assessment is given in Table 1.

**Table 1:** Risk of bias assessment.

**Reference**	**Random sequence generation**	**Allocation concealment**	**Blinding of participants and personnel**	**Blinding of outcome assessment**	**Incomplete outcome data**	**Selective reporting**	**Overall risk of bias**
Levin et al. (26)	Low risk	Low risk	Low risk	Low risk	Low risk	Low risk	Low risk
Levin et al. (27)	Low risk	Low risk	Low risk	Low risk	High risk	Low risk	Low risk
Hill et al. (28)	Low risk	High risk	High risk	High risk	High risk	Low risk	High risk
Allsop et al. (29)	Low risk	High risk	Low risk	Low risk	Low risk	High risk	High risk
Trigo et al. (30)	Low risk	Low risk	Low risk	Low risk	Low risk	Low risk	Low risk
Lintzeris et al. (31, 32)	Low risk	Low risk	Low risk	Low risk	High risk	Low risk	Low risk
Freeman et al. (33)	Low risk	Low risk	Low risk	Low risk	Low risk	Low risk	Low risk
D'Souza et al. (34)	Low risk	Low risk	Low risk	Low risk	High risk	Low risk	Low risk

## Results

### Study Selection

**Figure 1:**
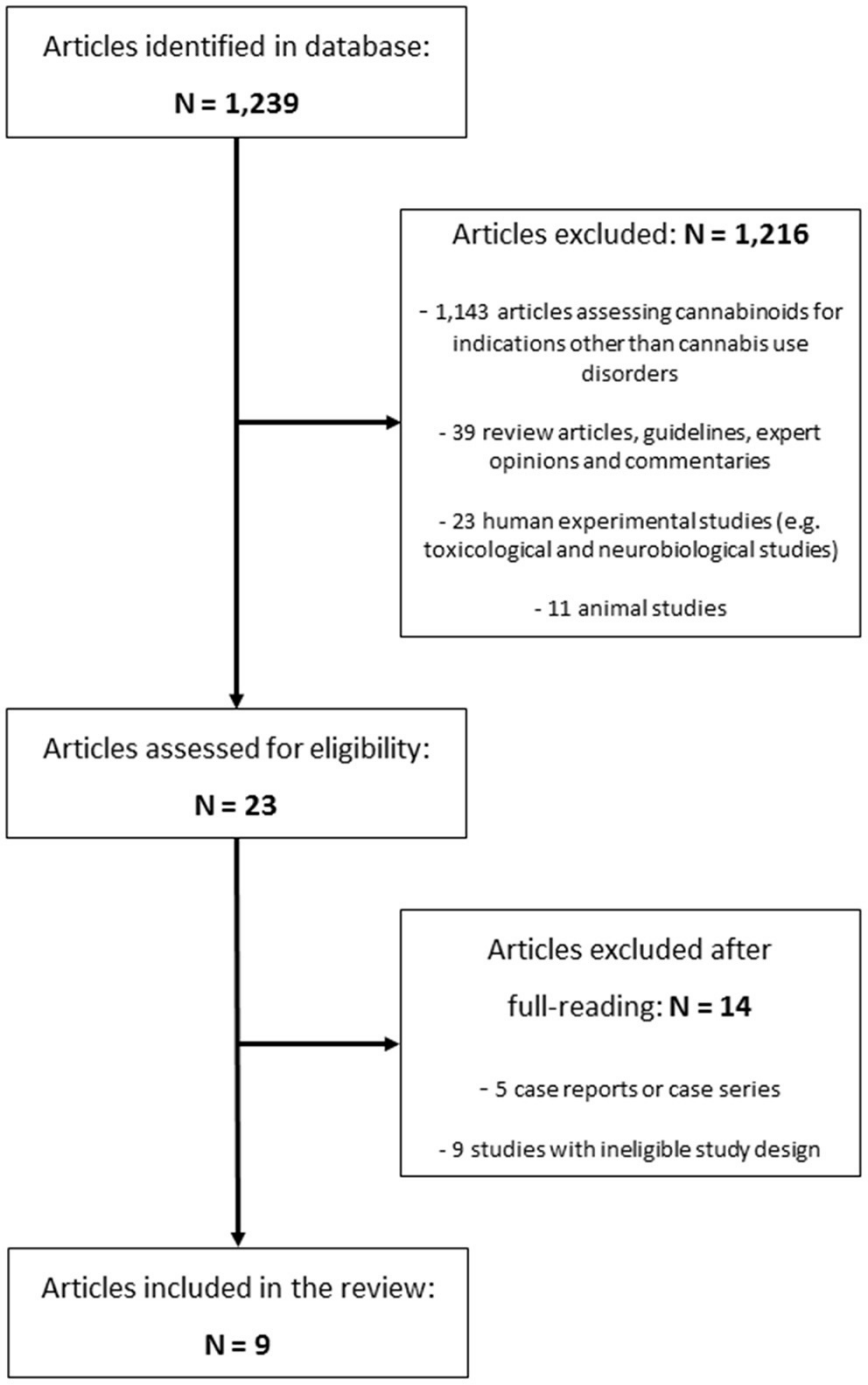
PRISMA flow diagram of study selection.

The systematic literature search revealed a total of 1,239 articles. After applying the above-mentioned eligibility criteria, 1,216 articles were excluded. In a second step, the two authors independently read the remaining 23 articles in full-text. Another 14 of the 23 articles were excluded because their study designs did not fulfill the criteria of a randomized controlled trial. The remaining nine articles met the final eligibility criteria (26–34). As two of the nine articles refer to one study (29/30), a total of eight studies was included to the systematic review. The corresponding PRISMA flow diagram is given in Figure 1.

### Study Characteristics

Eight studies with a total of 667 participants were included to this review. Four hundred and thirty five participants (65.2%) completed the studies according to the protocol. Although all studies met the final eligibility criteria, they varied widely in terms of population characteristics, study design, interventions and outcomes. The mean age of the participants ranged from 26.4 to 37.1 years with a male predominance of 73% across all studies. One study categorically included only male participants (34).

All studies were randomized, double-blind and placebo-controlled clinical trials with a parallel design. Seven of the eight studies were outpatient-only trials with a study duration ranging from 4 to 12 weeks. One of them had a 4-weekly assessment interval (29/30) and five of them had an assessment frequency of one to two times per week (26–28, 30, 33). The other two studies included an inpatient treatment phase of up to 9 days (29, 34). An adjunctive psychosocial therapy was applied to all participants in all trials during the active treatment period except for one (34). Two studies had a follow-up assessment after 4 weeks (28, 29) and one study after 12 weeks (29/30). One of the trials included multiple follow-up assessments up to week 24 (33). The remaining four studies had no follow-up assessments (26, 27, 30, 34).

All studies included participants with a CUD, as defined by the diagnostic criteria of the DSM-IV (27, 28, 30, 34), DSM-IV-TR (26, 29), DSM-V (33) and the International Statistical Classification of Diseases and Related Health Problems, 10^th^ edition (ICD-10) (29/30). Four of the eight studies included participants with a previous quit attempt, two of which needed to have presented withdrawal symptoms (29, 34) and the other two studies did not state the need for having experienced withdrawal symptoms (29/30, 34). The remaining four trials did not consider previous attempts to quit cannabis use (26–28, 30). Dependence of other substances (except for nicotine and caffeine) was excluded in all studies. Unstable or severe axis I mental disorders were excluded from seven trials (26–32, 34) and one study only excluded patients with psychotic disorders (33).

Cannabis use at baseline and, if allowed by the study protocol, during the study was reported in different ways. Four studies rated cannabis use according to the number of days of use ranging from 25.7 to 28 days within the 28 days prior to baseline (26–28, 31, 32). Two studies used the cannabis weight, ranging from 6.0 to 22.98 g per week at baseline (29, 30), and one study registered the number of joints, i.e., 3.7 joints per day at baseline (34). Only one trial did not provide any information on the frequency or amount of cannabis use at baseline (33).

With regard to the study interventions, the eight studies applied five different cannabinoid preparations: dronabinol (*n* = 2) (26, 27), nabilone (*n* = 1) (28), nabiximols (*n* = 3) (29/30, 31, 32), CBD (*n* = 1) (33), and PF-04457845, a novel FAAH inhibitor (*n* = 1) (34). One of the two dronabinol studies (27) included it's combination with lofexidine, an alpha-2 noradrenergic agonist used in the treatment of opioid withdrawal (35).

All trials were carried out in developed countries: four single-center studies in the United States (26–28, 34), two multi-center studies in Australia (29/30, 32), one single-center study in Canada (30) and one single-center study in the United Kingdom (33).

### Study Outcomes

The study characteristics and study outcomes are summarized in Tables 2, 3.

**Table 2:** Study characteristics.

**Reference**	**Country**	**N**	N*	**Males (%)**	**Age (y)**	**Intervention**	**Maximum dose**	**Study design**
Levin et al. (26)	USA	156	99	82.1	37.6	Dronabinol	40 mg/day	1-week outpatient placebo lead-in phase, followed by a 9-week treatment phase with a fixed dose schedule and a 2-week lead-out phase; no follow-up.
Levin et al. (27)	USA	122	67	68.8	35.1	Dronabinol + Lofexidine	Dronabinol 60 mg/day Lofexidin 1.8 mg/day	1-week outpatient placebo lead-in phase, followed by a 10-week treatment phase with a fixed-flexible dose schedule and a 1-week lead-out phase; no follow-up.
Hill et al. (28)	USA	18	12	66.7	26.4	Nabilone	2 mg/day	10-week outpatient treatment phase with a fixed dose schedule; follow-up after 4 weeks.
Allsop et al. (29)	Australia	51	35	76.5	35.4	Nabiximols	THC 86.4 mg/day CBD 80 mg/day	6-day inpatient treatment phase with a fixed dose schedule, followed by a 3-day washout phase; outpatient follow-up after 4 weeks.
Trigo et al. (30)	Canada	40	27	72.5	33.0	Nabiximols	THC 113.4 mg/day CBD 105 mg/day	12-week outpatient treatment phase with self-titrated study medication; target quit date for cannabis on day 21; no follow-up.
Lintzeris et al. (31, 32)	Australia	128	60	76.6	35.0	Nabiximols	THC 86.4 mg/day CBD 80 mg/day	12-week outpatient treatment phase with a 3-day dose induction period and weekly titrated doses; 12-week outpatient follow-up (*N* = 55).
Freeman et al. (33)	UK	82	77	72.0	26.4	Cannabidiol	A: 200 mg/day B: 400 mg/day C: 800 mg/day	4-week outpatient treatment phase with fixed doses; CBD 200 mg was stopped at interim analysis due to lack of efficacy; follow-up at weeks 6, 8, 12, 16, 20 and 24.
D'Souza et al. (34)	USA	70	58	100	28.2	PF-04457845	4 mg/day	5-(to 8)-day inpatient withdrawal phase, followed by a 3-week outpatient treatment phase with a fixed dose; no follow-up.

N, number of participants who were enrolled in the study. N^*^, number of participants who completed the study. THC, Δ^9^-tetrahydrocannabinol; CBD, cannabidiol.

**Table 3:** Main study outcomes.

**Reference**	**Intervention**	**Cannabis use**	**Abstinence**	**Withdrawal symptoms**	**Craving**	**Treatment retention**	**Adverse events**
Levin et al. (26)	Dronabinol	→	→	↓	N/A	↑	AEs: → SAEs: *N* = 4 (3 of them in the dronabinol group), not study-related
Levin et al. (27)	Dronabinol + Lofexidine	N/A	→	→	N/A	→	AEs: → SAEs: *N* = 2 (1 of them in the dronabinol + lofexidine group), not study-related
Hill et al. (28)	Nabilone	→	N/A	→	→	N/A	AEs: → SAEs: none
Allsop et al. (29)	Nabiximols	N/A	N/A	↓	↓	↑	AEs: → SAEs: *N* = 1 (in the placebo group)
Trigo et al. (36)	Nabiximols	→	→	→	→	N/A	AEs: → SAEs: none
Lintzeris et al. (31)	Nabiximols	↓	→	→	→	→	AEs: → SAEs: *N* = 1 (in the placebo group)
Lintzeris et al. (32)	Nabiximols	↓	↑	N/A	N/A	N/A	N/A
Freeman et al. (33)	CBD 200 mg CBD 400 mg CBD 800 mg	→ ↓ ↓	→ ↑↑	N/A → ↓	N/A N/A N/A	N/A N/A N/A	All doses: AEs: → SAEs: none
D'Souza et al., 2018	PF-04457845	↓	N/A	↓	N/A	N/A	AEs: → SAEs: none

*↑, significant increase compared to placebo; → , non-significant effect compared to placebo; ↓, significant reduction compared to placebo; N/A, not evaluated; AEs, adverse events; SAEs, serious adverse events; CBD, cannabidiol*.

#### Cannabis Use

*Dronabinol:* Dronabinol reduced self-reported cannabis use during an overall 12-week treatment phase compared to baseline. However, there were no differences in this aspect between the dronabinol and placebo group (26).

*Nabilone:* Nabilone had no effect on the magnitude of cannabis use compared to placebo (28).

*Nabiximols:* Nabiximols significantly reduced self-reported cannabis use during a 12-week treatment period compared to placebo (31). This finding remained significant at the week-24 follow-up after ceasing the treatment (32). However, another study did not find any between-group difference during a 12-week treatment phase (30) and a fourth study did not assess the use of cannabis as an outcome variable (29).

*Cannabidiol:* CBD 400 mg and 800 mg per day were more efficacious than placebo at reducing cannabis use during a 4-week treatment phase compared to placebo, as confirmed by decreased urinary THC-COOH:creatinine ratios (33). The reductions in cannabis use were maintained up to the final follow-up (week 16) in the CBD 400 mg group, but not in the 800 mg group.

*FAAH inhibitor:* PF-04457845 significantly reduced self-reported cannabis use during a 4-week treatment phase compared to placebo, as confirmed by reduced urinary THC-COOH concentrations (34).

#### Abstinence

*Dronabinol:* Dronabinol did not differ from placebo in the proportion of study participants who achieved two consecutive weeks of abstinence at the end of an 8-week maintenance phase (26). Dronabinol in combination with lofexidine also failed to demonstrate any difference in the proportion of participants with 3 weeks of abstinence during a 6-week maintenance phase compared to placebo (27).

*Nabilone:* not evaluated.

*Nabiximols:* One study reported no significant difference in the number of participants who have achieved a period of abstinence from cannabis of at least 4 weeks during a 12-week treatment phase with nabiximols compared to placebo (31). However, there was a significantly higher proportion of participants of the nabiximols group than the placebo group of the same study sample who reported abstinence in the previous 4 weeks at the week-24 follow-up (32). In another study, nabiximols did not differ from placebo regarding abstinence rates at the end of a 12-week treatment phase (30). A fourth study did not evaluate abstinence rates (29).

*Cannabidiol:* CBD 400 mg and 800 mg increased the number of days per week with abstinence from cannabis during a 4-week treatment phase compared to placebo, as assessed by self-reports (33).

*FAAH inhibitor:* not evaluated.

#### Withdrawal

*Dronabinol:* Dronabinol showed a greater reduction of cannabis withdrawal symptoms during an overall study period of 12 weeks, compared to placebo (26). On the other hand, dronabinol combined with lofexidine showed no significant effect on weekly cannabis withdrawal scores during a 10-week study period (27).

*Nabilone:* Nabilone did not differ from placebo in the reduction of cannabis withdrawal symptoms during a 10-week treatment period (28).

*Nabiximols:* One study found a significant decrease in withdrawal symptoms by nabiximols during a 6-day treatment phase compared to baseline, while the withdrawal scores in the placebo group increased (29). Moreover, the duration of the withdrawal syndrome was shorter and the peak of symptoms occurred earlier. Two studies reported a reduction of withdrawal symptoms during the treatment with nabiximols (up to 12 weeks), but without any significant differences between nabiximols and placebo (30, 31).

*Cannabidiol:* CBD 800 mg, but not 400 mg, was more efficient in reducing cannabis withdrawal symptoms during a 4-week treatment phase and follow-up, compared to placebo (33).

*FAAH inhibitor:* PF-04457845 significantly reduced symptoms of cannabis withdrawal during about a week of forced abstinence, compared to baseline and placebo (34). Consistently, the PF-04457845 group also reported less depression, irritability, anxiety and sleep disturbances which are symptoms likely related to cannabis withdrawal.

#### Craving

*Dronabinol:* not evaluated.

*Nabilone:* Nabilone and placebo reduced cannabis craving during a 10-week treatment phase. However, there were no significant treatment group differences at either the end of treatment or the end of a 4-week follow-up period (28).

*Nabiximols: One* study reported a significantly greater reduction of cannabis craving during a 6-day treatment episode in the nabiximols group compared to the placebo group (29). Two studies reported a reduction of cannabis craving during the treatment with nabiximols and placebo (up to 12 weeks), but with no significant between-group differences (30, 31).

*Cannabidiol:* not evaluated.

*FAAH inhibitor:* not evaluated.

#### Retention in Treatment

*Dronabinol:* The retention in treatment at the end of an 8-week maintenance phase was significantly higher in the dronabinol group compared to the placebo group (26). However, the combination of dronabinol and lofexidine showed no difference from placebo in the retention rate at the end of a 6-week maintenance phase (27).

*Nabilone:* not evaluated.

*Nabiximols:* Nabiximols was associated with a higher rate of treatment retention at the end of a 6-day medication phase compared to placebo (29). Another study could not confirm a difference between the nabiximols and the placebo group regarding the retention in treatment (31), and one study did not report on this outcome variable (30).

*Cannabidiol:* not evaluated.

*FAAH inhibitor:* not evaluated.

#### Adverse Events

*Dronabinol:* The maximum dose of 40 mg per day was well tolerated with no differences between dronabinol and placebo regarding the number of adverse events (26). Four serious adverse events were reported (hospitalization because of worsening of diabetes, worsening of chronic asthma, stomach virus and altercation with the police), three in the dronabinol and one in the placebo arm, which were not deemed to be study-related. Similarly, there were no significant differences between dronabinol in combination with lofexidine and placebo regarding the overall number of adverse events (27). There were two serious adverse events (hospitalization because of abdominal pain, admission to a detoxification program), one in each study arm, which were not considered to be related to the study procedure.

*Nabilone:* Doses of 2 mg were well-tolerated. All reported adverse events were mild to moderate and no serious adverse event was recorded (28).

*Nabiximols:* No between group differences in adverse events were reported (29–31). In the three studies, two serious adverse events occurred, each in the placebo group [hospitalization for suicidal ideation (31) and threat of suicide (29)].

*Cannabidiol:* CBD at 400 mg and 800 mg per day was well tolerated. There was no difference in the number of mild or moderate adverse events between both dosage groups and the placebo group. No serious adverse events were recorded (33).

*FAAH inhibitor:* PF-04457845 was well tolerated. The recorded adverse events were mild and the number of adverse events did not differ from the placebo group. There were no serious adverse events and the FAAH inhibitor did not influence the dropout rate (34).

## Discussion

This study aimed to systematically review the current literature on the use of cannabinoids in the treatment of CUD and to summarize the main findings in terms of efficacy, safety and tolerability provided by randomized controlled trials. We identified eight studies which examined the effects of five cannabinoid preparations on specific clinical outcome variables, i.e., cannabis use, abstinence, withdrawal, craving, retention in treatment and adverse events. Regarding their, at least in part, different mechanisms of action, we could classify the cannabinoids to two therapeutic strategies: (A) agonist substitution and (B) endocannabinoid modulation.

The agonist substitution therapy, also known as replacement therapy, has been proven to be effective in various substance use disorders, particularly in nicotine and opioid dependence. It therefore appeared obvious to test cannabinoid receptor agonists in the treatment of CUD. In this respect, three cannabinoid preparations were of particular interest, dronabinol, nabilone, and nabiximols. Dronabinol and nabilone are currently used as a second line treatment for patients with AIDS/cancer cachexia and for chemotherapy patients experiencing nausea or vomiting (37, 38). Nabiximols is used in the treatment of central neuropathic pain in multiple sclerosis and as an adjuvant analgetic in adults with advanced malignancy (36). In several human laboratory studies, CB1 receptor agonists have been shown to alleviate symptoms of cannabis withdrawal and to reduce relapse in patients with CUD (39, 40).

As expected, dronabinol attenuated cannabis withdrawal symptoms and improved retention in treatment but failed to reduce cannabis use and to improve abstinence, which was the primary outcome of the two studies (26, 27). It was suggested that the CB1 receptor-agonistic properties of dronabinol successfully counteracted the development of withdrawal symptoms, but the low motivation to quit among the participants might have been responsible for the lack of an effect on cannabis use and abstinence. The authors therefore concluded that the participants would have benefited from a longer maintenance period in order to better promote a motivation for sustainable change. Similarly, nabiximols improved withdrawal symptoms, craving and treatment retention, but, in contrast to dronabinol, also reduced cannabis use and improved abstinence (29, 31, 32). This significant difference from dronabinol in the therapeutic profile might be explained by the additional presence of CBD for which “anti-addictive” action has been described recently, see further below (41). On the other hand, nabilone failed to demonstrate any beneficial effects on cannabis use, withdrawal or craving. In this context, the authors speculated whether the dose of nabilone might have been too low in order to display therapeutic efficacy in CUD (28). Moreover, the sample size of 18 participants of whom only 12 completed the overall treatment phase seems too small to draw any robust conclusion on nabilone's efficacy.

The modulation of the endocannabinoid system is a relatively novel approach in the treatment of CUD (22). The endocannabinoid system consists of specific cannabinoid receptors, i.e., the CB1 and CB2 receptor, their primary endogenous ligands AEA and 2-AG, and the AEA- and 2-AG-degrading enzymes FAAH and monoacylglycerol lipase (MAGL) (20, 42). At the molecular level, endocannabinoids play a crucial role in the regulation of various neurotransmitter systems, including the dopaminergic mesolimbic reward pathways (43). It is therefore suggested that the modulation of the endocannabinoid system might have the potential to normalize the dopamine signaling which is typically disrupted by heavy cannabis use, and, thus, appear to be a promising target in the treatment of CUD.

In this context, CBD, the second most abundant constituent of cannabis, is a cannabinoid of substantial interest with regard to the treatment of CUD (41). CBD has only minimal direct action at cannabinoid receptors but primarily acts as an inhibitor of the hydrolysis and reuptake of endocannabinoids (44) as well as a negative allosteric modulator of the CB1 receptor (45), thereby counteracting the acute psychoactive effects of THC (46). As a modulator of the endocannabinoid system, CBD reduced cannabis use, improved abstinence and attenuated withdrawal symptoms (33). The reductions in cannabis use at week-16 follow-up was only evident in the 400 mg-group and the reductions in cannabis withdrawal was only evident in the 800 mg-group. In this case, it can be assumed that the enhancement of the endocannabinergic activity might have contributed to the beneficial effects of CBD.

Another approach for the treatment of CUD also referred to the modulation of the endocannabinoid system by the inhibition of FAAH. In this context, the selective FAAH inhibitor URB597 has been reported to attenuate withdrawal symptoms in an animal model of CUD by increasing AEA concentrations (47). Moreover, mice with reduced FAAH expression due to a genetic variation have been shown to be less likely to develop CUD than the wild-type carriers (48). PF-04457845 is a novel and highly selective and potent human FAAH inhibitor which, so far, was mainly tested in patients with diverse pain syndromes (49). The only study in the present review which evaluated the efficacy of PF-04457845 in CUD showed reduced cannabis use and cannabis withdrawal symptoms compared to placebo (34). The authors suggested that the increase of endocannabinoid concentrations by selective FAAH inhibition might have been the key mechanism contributing to this outcome.

## Limitations

The results of the studies summarized in this review need to be interpreted with caution. First of all, the studies showed a large variety, particularly with regard to population characteristics (low motivation vs. high motivation to quit cannabis use), study setting (inpatient vs. outpatient), study design (forced abstinence vs. harm reduction), concomitant interventions (psychosocial therapy vs. no psychosocial therapy) and operationalization of outcome measures (e.g., cannabis use as assessed by the frequency or the amount of use). These differences impede a meaningful comparison of the efficacy of different cannabinoids. Second, the drop-out rate of about one third among all studies was relatively high which might have affected the significance of the respective study results. Third, specific populations were underrepresented, such as women, older people and individuals from different ethnicities or with comorbid mental disorders or other substance use disorders. In this respect, the study results are less generalizable to other populations and do not picture the real world. Finally, the effects of the different cannabinoids on the various outcome variables were rather modest, most probably due to the relatively small sample sizes.

## Conclusions

The agonist substitution (replacement) approach with the CB1 receptor agonist dronabinol showed efficacy in the reduction of cannabis withdrawal symptoms but it was not able to demonstrate an influence on cannabis use or abstinence. In contrast, the modulation of the endocannabinoid system by CBD or the selective FAAH inhibitor PF-04457845 seems to be efficacious for both reducing withdrawal symptoms and improving cannabis use and abstinence. As endocannabinoid modulators, compared to CB1 receptor agonists, also produce lower abuse liability and less intoxication, they appear to be a promising group of drugs for the treatment of CUD (22). However, the evidence is at this time too weak to support any specific medication. Future studies should include greater sample sizes, more diverse populations, longer treatment periods and head-to-head comparisons.

## Data Availability Statement

The original contributions presented in the study are included in the article/supplementary material, further inquiries can be directed to the corresponding author.

## Author Contributions

PR designed the study. CV and PR performed the literature searches, screened the studies, and wrote the first draft of the manuscript. CV, NS, UB, and PR critically contributed to the discussion and approved the final version of the article. All authors contributed to the article and approved the submitted version.

## Funding

The publication of this study was supported by the Open Access Publication Fund of the University of Duisburg-Essen.

## Conflict of Interest

The authors declare that the research was conducted in the absence of any commercial or financial relationships that could be construed as a potential conflict of interest.

## Publisher's Note

All claims expressed in this article are solely those of the authors and do not necessarily represent those of their affiliated organizations, or those of the publisher, the editors and the reviewers. Any product that may be evaluated in this article, or claim that may be made by its manufacturer, is not guaranteed or endorsed by the publisher.
